# Phenotypic differentiation and diversifying selection in populations of *Eruca sativa* along an aridity gradient

**DOI:** 10.1186/s12862-022-01996-w

**Published:** 2022-03-30

**Authors:** Prabodh Kumar Bajpai, Harel Weiss, Gony Dvir, Nir Hanin, Haggai Wasserstrom, Oz Barazani

**Affiliations:** Institute of Plant Sciences, Agricultural Research Organization-Volcani Institute, 7505101 Rishon LeZion, Israel

**Keywords:** Aridity, Genetic differentiation, Local adaptation, Phenology, Phenotypic variation, Precipitation, Selection

## Abstract

**Background:**

The aridity gradient in the eastern Mediterranean offers an opportunity to investigate intra-specific genetic differentiation and local adaptation in plant populations. Here we used genetic (F_ST_) and quantitative trait (P_ST_) differentiation to assess local adaptation among three natural populations of *Eruca sativa* (Brassicaceae) distributed along a climatic range representing desert, semi-arid and Mediterranean habitats.

**Results:**

Amplified fragment length polymorphism (AFLP) analysis revealed high genetic diversity in each population, but low genetic differentiation between populations and relatively high gene flow. Further phenotypic evaluation in a common garden experiment (conduced in a Mediterranean habitat) showed clear differences in phenological traits among populations (day of flowering and duration of the reproductive stage), shoot and root biomass, as well as fitness-related traits (total number of fruits and total seed weight). F_ST_–P_ST_ comparison showed that P_ST_ values of the phenological traits, as well as below- and above-ground biomass and fitness-related traits, were higher than the F_ST_ values.

**Conclusions:**

Overall, our results support the identification of genotypic and phenotypic differentiation among populations of *E. sativ*a. Furthermore, the F_ST_–P_ST_ comparison supports the hypothesis that these were subjected to past diversifying selection. Thus, the results clearly demonstrate adaptive divergence among populations along an aridity gradient, emphasize the ecological value of early flowering time in arid habitats, and contribute to our understanding of the possible impact of climate change on evolutionary processes in plant populations.

**Supplementary Information:**

The online version contains supplementary material available at 10.1186/s12862-022-01996-w.

## Background

Adaptation to local environmental conditions—a perquisite for a plant’s long-term survival in any geographic region—requires intra-species variation in life-history traits [1–4]. Indeed, studies that investigated phenotypic differentiation covering a wide range of environmental conditions and spatial scales, have shown that variation in flowering time in both annual and perennial species is influenced by temperature [5–7] and precipitation gradients [8–10]. In addition, results of a study that tested root and shoot biomass of 239 species across a range of 1874 sites showed distinct patterns of plants allocating resources towards below-ground biomass in stressful environments—clear evidence for the environmental effect on plant growth strategies [11]. The adaptive value and significance of these types of trait differentiation can be determined by testing local selection processes. In addition, the study of intraspecific adaptation along environmental gradients provides opportunity to identify local conditions supporting or resisting adaptive consistency [12].

Phenotypic differentiation in quantitative traits can be caused either by selection or random genetic drift, but distinguishing between the two is often difficult [13]. A common but indirect approach to evaluate whether local adaptation has shaped genetic variation is to compare neutral genetic differentiation (F_ST_) with quantitative trait differentiation (Q_ST_) assessed in common garden conditions [4, 8, 14, 15]. Alternative approaches to infer past selection also include phenotype or genotype based inference [16]. The first, is mainly limited to organisms having fast generation time and involves experimental systems for direct measurement of neutral divergence, such as mutation-accumulation and estimated time since divergence; estimations that usually are not easily available. The genotype-based approach is generally based on McDonald–Kreitman test or genome scan-outlier loci methods. However, these methods cannot be correlated with phenotypic traits, thus limiting their application when assessing the adaptive value of a trait of interest. The Q_ST_–F_ST_ approach thus offers better method for gauging past selection by providing the opportunity to link genetic differentiation with phenotypic traits. In addition, Q_ST_ reaches equilibrium faster than allele frequencies at a specific QTL (quantitative trait loci) (tens rather than hundreds of generations), thus offering an advantage over genome scans. Furthermore, the Q_ST_ approach is more straightforward in comparison to other natural selection prediction methods where null (neutral) distributions are dependent on multiple assumptions [16].

The Q_ST_–F_ST_ approach has been extensively and effectively used in analyzing adaptation to local environmental conditions in 13 populations of two *Antirrhinum majus* subspecies distributed along an elevation gradient in Southern France [12]. This approach also demonstrated diversifying selection for phenology differentiation among nine populations of *Silene ciliata* in Spain distributed along elevation and temperature gradients [17], and for fitness-related traits among European populations of *Campanula rotundifolia* along varied climatic (temperature and precipitation) conditions [8]. Geographic and climatic gradients therefore offer a valuable framework to assess ecotypic differentiation in plant species and to identify corresponding adaptive traits [18, 19].

The steep aridity gradient in the eastern Mediterranean creates varied ecological clines over relatively short distances. Widely distributed species in this region, exhibiting signatures of inter-population genetic differentiation of life history traits, can thus provide insights into the evolutionary processes that led to local adaptations. In this geographical range of increasing aridity to the south and east, the major limiting factor for plant growth and seed production is exposure to variable or unpredictable amounts of rainfall [20]. Indeed, several studies demonstrated clear associations between climatic conditions in the eastern Mediterranean and flowering time of widely distributed species [10, 21–25], pointing to the ecological importance of early flowering in ensuring plant fitness in arid environments. However, as far as we know, only two studies in this region employed F_ST_–Q_ST_ analysis in the investigation of local adaptations, specifically several growth and reproductive life history traits among populations of *Hordeum spontaneum* [4, 26].

**Fig. 1:**
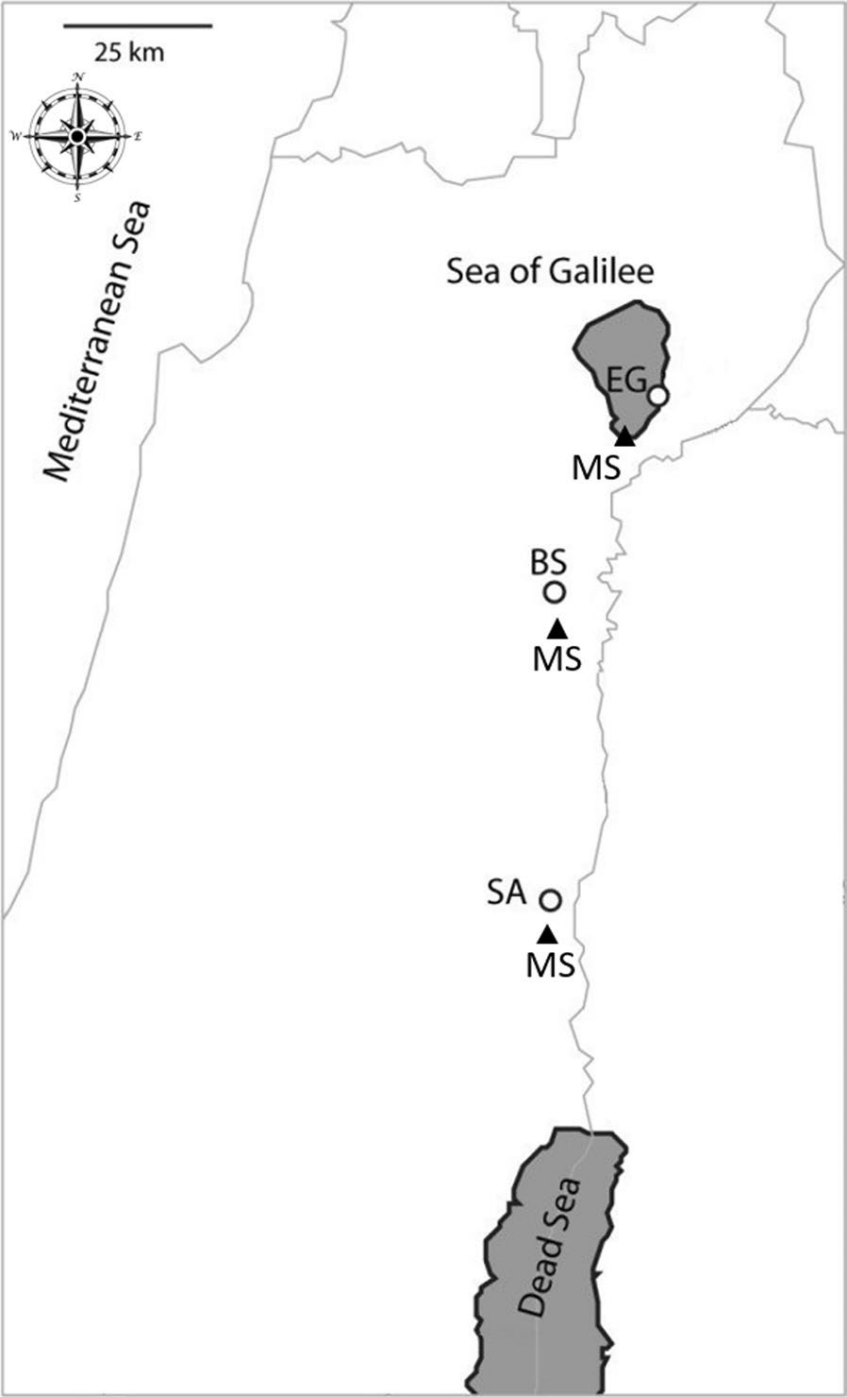
Geographical locations of three populations in the eastern Mediterranean: Sartaba (SA), Bet Shean (BS) and Ein Gev (EG). The locations of nearest metrological station (MS) from which climatic information was gathered is also presented

*Eruca sativa* Mill. (Brassicaceae) is an insect-pollinated self-incompatible winter annual species [27]. In the eastern Mediterranean, populations of *E. sativa* are distributed along a narrow geographical range from the southern Golan Heights in the north, to the Jordan Valley in the south, across a steep north–south climatic gradient changing from mesic Mediterranean to arid-hot desert conditions [20, 25]. We previously showed phenological and phenotypical variations among populations of *E. sativa* along this aridity gradient [25, 28–30]. More recently, we also reported on genetic differentiation and additive genetic variance for flowering time plasticity in response to the plant hormone jasmonate in these populations [31]. To gain more insight into the evolutionary responses that led to ecotypic differentiation, in this study we investigated whether variation in phenological and associated fitness-related traits in *E. sativa* populations have been under past selection. To this aim, we investigated three populations across an aridity gradient of Mediterranean, semi-arid and desert habitats (Fig. 1 and Additional file 1: Table S1). These populations represent the main different genetic clusters of *E. sativa* along the investigated geographical range [25]. A combined approach of molecular marker differentiation with quantitative trait differentiation analysis, conducted in a common garden experiment, helped us infer differentiation in adaptive traits.

## Results

### Genetic diversity and genetic differentiation among populations

Amplified fragment length polymorphism (AFLP) was selected as the marker of choice for this study, as it provides robust polymorphism with no prior genomic information, and thus offering a relatively quick mean to understanding genetic variation. The analysis, with six different primer combinations, yielded 90 loci that could be reliably scored (Additional file 2), of which 84% were polymorphic at the 93.33% level.

Overall, the genetic diversity parameters (number of polymorphic loci, percentage of polymorphic loci, Nei’s gene diversity and Shanon information index) were relatively high in all three populations (Table 1). The semi-arid population (Bet Shean, BS) showed low genetic diversity as compared to the desert (Sartaba, SA) and Mediterranean (Ein Gev, EG) populations. However, these differences were not significant (see Table 1). Further analysis of molecular variance (AMOVA) showed overall high variance within populations (15.022) in comparison to the variance among populations (1.211) (Additional file 1: Table S2). The overall genetic differentiation (F_ST_ = 0.04) among populations was relatively low [32], which was evident from the high level of gene flow (12.50) (Table 1).

**Table 1:** Genetic diversity values of the three populations of *E. sativa*; 95% upper and lower confidence intervals (CI) are also provided in brackets

	N	NPL	PPL (%)	H^a^ (CI)	I^a^ (CI)	F_ST_ (CI)	Gene flow
Ein Gev	22	86	95.56	0.3542 ± 0.1449 (0.3239–0.3846)	0.5238 ± 0.1869 (0.4846–0.5629)	0.04 (− 0.0083–0.0077)	12.50
Bet Shean	27	82	91.11	0.3378 ± 0.1609 (0.3041–3715)	0.4994 ± 0.2138 (0.4547–0.5442)		
Sartaba	24	84	93.33	0.3478 ± 0.1513 (0.3161–0.3795)	0.5142 ± 0.1984 (0.4726–0.5557)		
Average		84	93.33	0.3466 ± 0.0083 (0.3284–0.3648)	0.5125 ± 0.0123 (0.4886–0.5364)		

NPL: number of polymorphic loci; PPL: percentage of polymorphic loci; H: Nei’s gene diversity; I: Shanon information index

^a^Expressed as mean ± standard deviation

### Phenotypic variation among populations of *E. sativa*

Phenotypic evaluation was assessed in two common garden experiments conducted in field and net-house conditions, respectively. At the field site we monitored phenological traits from the onset to the end of the reproduction phase. Potted plants in the net-house enabled us to determine the biomass of roots and shoots, and assess fitness-related traits (fruit set and seed mass), as all plants in the field gained substantial masses.

**Fig. 2:**
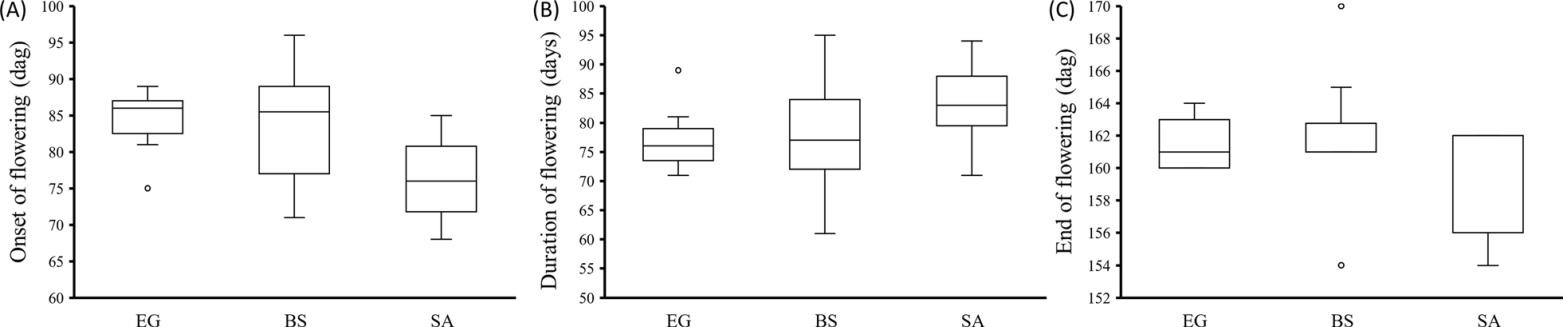
Box-plot showing variation in phenology traits in the three *E. sativa* populations (N = 20 for each population): **A** Onset of flowering, **B** Duration of flowering, and **C** End of flowering. Onset and end of flowering are presented as days after germination (dag). Duration of flowering is presented as days between onset of flowering and end of flowering; (○) presents outlier values

**Fig. 3:**
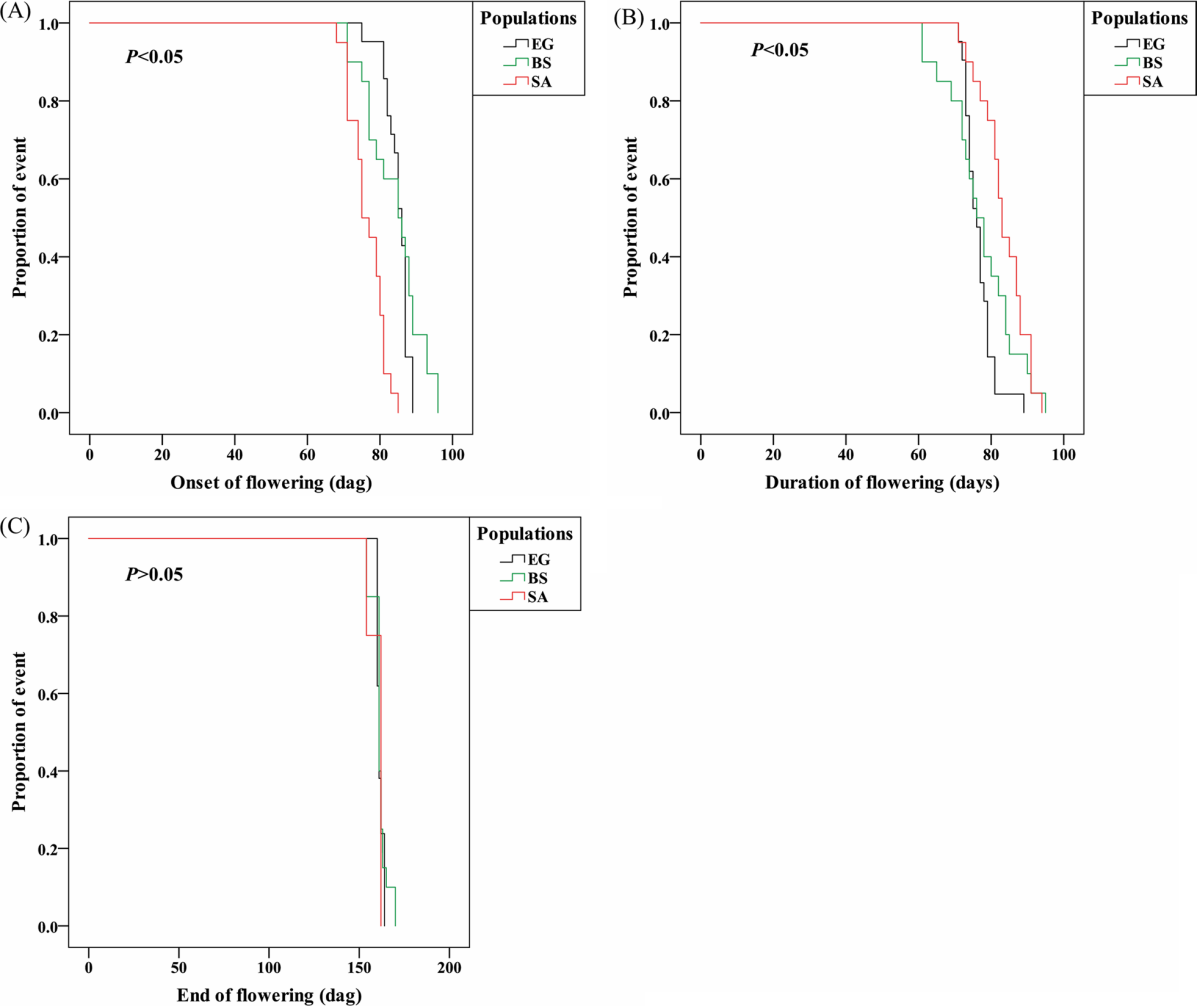
Kaplan–Meier non-parametric survival curves and *P* values (based on the Log-Rank test) comparing flowering phenology traits in three populations of *E. sativa*: **A** Onset of flowering, **B** Duration of flowering, and **C** End of flowering. Onset and end of flowering are presented as days after germination (dag). Duration of flowering is presented as days between onset of flowering and end of flowering

Results of the common garden field experiment showed that among the three populations, plants of the SA desert population showed the earliest onset of flowering (68–85 days). Plants of the Mediterranean population (EG) showed a narrower variation in the onset of flowering (81–89 days, excluding a single outlier) and duration of flowering (71–81 days, excluding the outlier); the widest variation in these two traits were found in plants of the BS semi-arid population (71–96 and 61–95 days, respectively) (Fig. 2). The end of flowering ranged between 154–162 days after germination in plants of the SA population, while variations in the other two populations were comparatively smaller (Fig. 2). A further log-rank test applied to Kaplan–Meier survival curves (Fig. 3 and Additional file 1: Table S3) revealed that day of flowering and the duration of flowering significantly differed among the three populations (χ^2^ = 13.68, *P* < 0.05 and χ^2^ = 10.89, *P* < 0.05, respectively), while no significant difference was found in the day when flowering ended (χ^2^ = 0.07, *P* > 0.05).

**Fig. 4:**
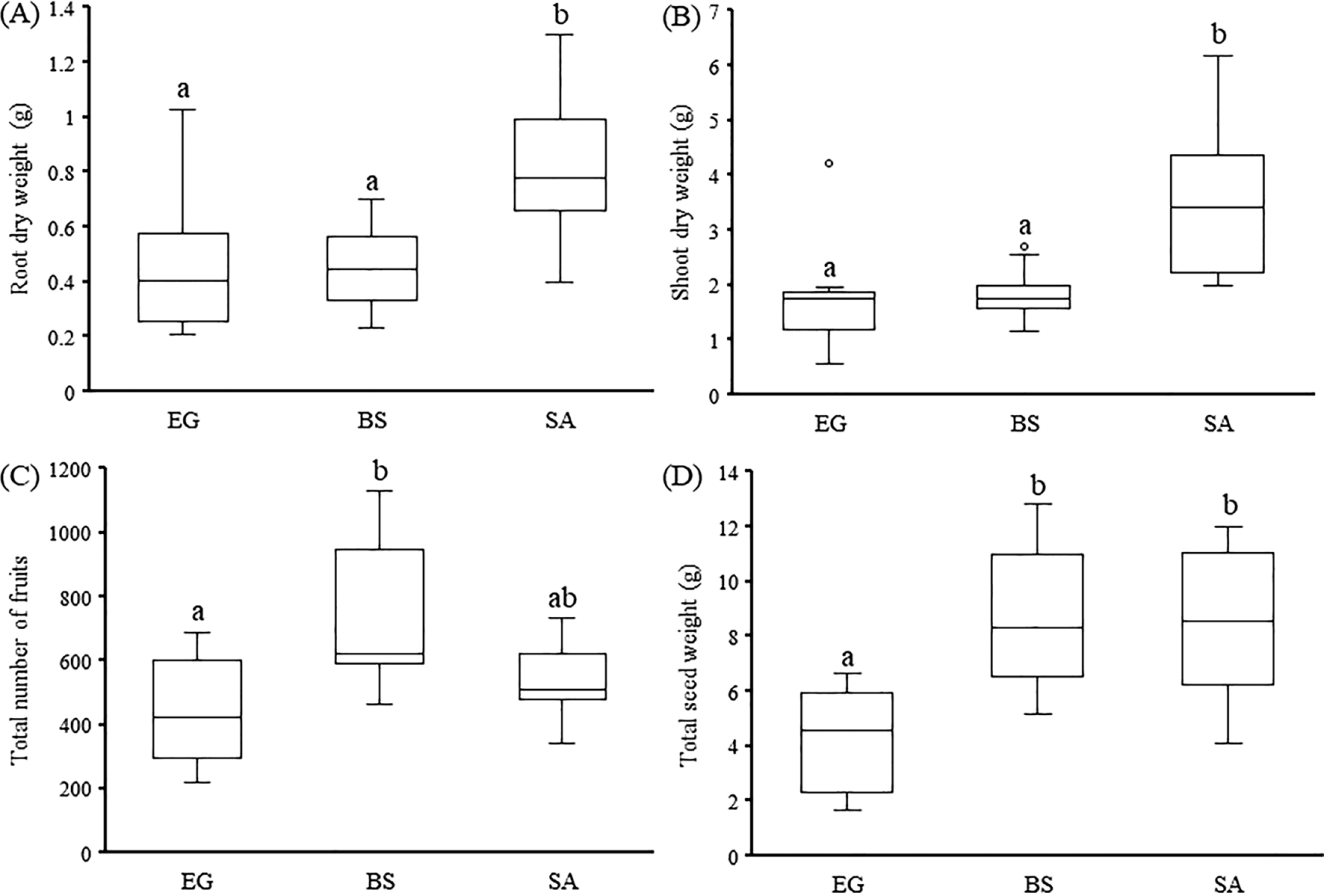
Box-plot showing variation in plant fitness related traits in the Mediterranean (EG), semi-arid (BS) and desert (SA) populations of *E. sativa* (N = 10 for each population): **A** Root dry weight (g); **B** Shoot dry weight (g); **C** Total number of fruits; and **D** Total seed weight (g); (○) presents outlier values. Different letters above each box represent significant differences (ANOVA Tukey post-hoc range test, *P* < 0.05)

Other phenotypic characteristics, i.e., plant biomass, the total number of fruits and total seed weight, were assessed in the potted net-house experiment. Plants of the desert population (SA) produced significantly higher shoot (*F* = 15.48, *df* = 2, *P* < 0.05) and root (*F* = 14.98, *df* = 2, *P* < 0.05) dry biomass as compared to plants of the other two populations (Fig. 4). No significant difference (*F* = 0.467, *df* = 2, *P* = 0.63) was found between plants of the three populations in root to shoot ratio, but the total number of fruits per plant (*F* = 6.57, *df* = 2, *P* < 0.05) differed significantly among plants of the three populations (Fig. 4). Consequently, the total seed weight per plant (*F* = 11.88, *df* = 2, *P* < 0.05) was significantly higher in plants of semi-arid and desert populations than in plants of the Mediterranean population (Fig. 4).

### Environmental data

Information gathered from meteorological stations located in geographical vicinity of the investigated populations provides information about the climatic gradient in the 20 years preceding seed collection (Additional file 1: Fig. S1). The data from these sites indicate a trend of declining rainfall and increasing average winter temperature in the Mediterranean and semi-arid habitats, while an opposite trend in rainfall is observed in the desert habitat.

### Assessing past selection

**Fig. 5:**
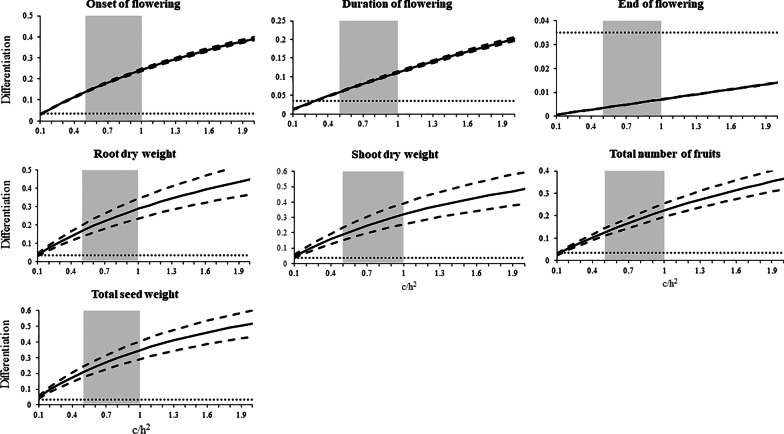
P_ST_ (solid line) and F_ST_ (dotted line) graphical comparisons for phenotypic traits in the three *E. sativa* populations. The dashed line indicates the P_ST_ 95% confidence intervals, and the grey area indicates the probable range for c/h^2^ between 0.5 and 1.0

Comparisons between neutral genetic differentiation (F_ST_) and quantitative trait differentiation (Q_ST_) are widely used to test for past selection on traits [8, 14, 15]. In our study we lacked the necessary kinship information, i.e., phenotypic measurements in half-sib or full-sib families, (e.g. [4]) for Q_ST_. However, as suggested by Leinonen et al. [33], Q_ST_ can be estimated using the P_ST_ approximation. The P_ST_ values of each of the phenotypic measurements, i.e., phenological traits (onset of flowering, its duration, and the end of the flowering stage), root and shoot biomass, as well as fitness-related traits (total average number of fruits and total seed weight) were calculated and compared to the obtained F_ST_ values. The results showed that the P_ST_ of the onset and duration of flowering exceeded the molecular marker differentiation (F_ST_) for the *c/h*^2^ range of 0.5–1.0 (i.e., P_ST_ > F_ST_, diversifying selection) (Fig. 5 and see more information on *c/h*^2^ in “Methods”). However, the P_ST_ value for the end of flowering was lower than F_ST_, indicating a stabilizing selection of this trait. All fitness-related traits, below- and above-ground biomass, total number of fruits, and total seed weight per plant showed higher P_ST_ values in comparison to F_ST_.

## Discussion

The common garden experiments clearly demonstrated phenotypic differentiation among populations of *E. sativa* along their distribution range over a gradient of climatic conditions and ecological pressures [25, 31]. Early flowering differentiated the desert population of *E. sativa* (SA) from populations from semi-arid and Mediterranean habitats with more favorable conditions (BS and EG). Similar results were obtained when plants of various populations of *E. sativa* were grown from seeds lots that were produced under similar conditions [25]. Thus, by reducing the possibility of maternal effect, the results suggested on genetic differentiation in phenological traits. Moreover, we previously reported additive genetic variation in arid and Mediterranean populations of *E. sativa* [31]. Thus, all together, our results indicate evolutionary divergence between populations of *E. sativa* along the species distribution range in the east Mediterranean.

It is accepted that an earlier transition to reproduction is a stress avoidance strategy associated with increasing aridity, one that reduces the probability of reproductive failure during a shorter life cycle in arid conditions [23, 34]. Thus, our results support previous studies demonstrating the eco-physiological role of early flowering in annual populations at arid sites (e.g., [21, 23, 24, 35, 36]). In addition, plants of the more arid populations (SA and BS) showed substantially higher variation in phenological traits compared to plants of the Mediterranean population. Therefore, our results support the marginal-central concept, suggesting that harsh and unpredicted climatic conditions favor the accumulation of diversity within a population [37].

Despite the expected shorter duration of flowering with increasing aridity [38–40], the reproduction phase of the desert population (SA) was longer than plants from more humid environments. It is possible that the longer duration of flowering in plants of the desert population reflects the relatively favorable conditions in the common garden experiment. However, Pau et al. [41] emphasized the relative importance of biotic drivers that change over the growing season to the shaping of phenological traits. Thus, the relative role of plant community structure, herbivore pressure and pollinator availability that differentiate the desert and Mediterranean populations of *E. sativa* [25, 28, 31] cannot be ruled out. Nevertheless, the differences between flowering duration in the three populations led to almost twice the reproductive biomass (total seed weight per plant) of the arid and semi-arid populations (SA and BS) in comparison to the Mediterranean population. It was previously suggested that in more productive environments plants need to invest more in vegetative growth to withstand higher neighbor densities and competition [36, 42], whereas plants from arid environments can allocate more of their above ground biomass to seed production [22, 36, 42, 43]. Accordingly, it is expected that in arid habitats, resource allocation towards root growth would be higher in comparison to above-ground biomass, and vice versa in more mesic environments [44, 45]. In contrast to the classic theory of optimal resource partitioning, no significant differences were found in root to shoot biomass among the plants of the three populations. However, this could be the result of the relative favorable conditions in the common garden experiment, which may have an impact on plastic response of root traits [46].

AFLP analysis provided a robust estimation of genetic parameters that showed high genetic diversity in populations of *E. sativa*, which were further substantiated by the high molecular variance within the populations, and low F_ST_ values. Although there was significant gene flow between populations, our results show that most of the measured phenotypic traits were subject to past diversifying selection. Similarly, Volis et al. [4] demonstrated adaptive genetic differentiation in several life history traits (e.g., number of spikelets, tiller height, leaf length and width, etc.) among 20 populations of *H. spontaneum* in the eastern Mediterranean. It is generally believed that gene flow and local selection pressures are opposite effects which can counteract each other [47–49]. Thus, it is reasonable to assume that phenotypic adaptive divergence among populations of *H. spontaneum* can be linked to the limited gene flow in this self-pollinated species. However, our results, showing genetic differentiation in a self-incompatible species within considerably short geographical distances between populations [25], support previous studies demonstrating the importance of environmental factors as divergent and effective selective pressures that shape phenotypic differentiation [17, 50]. Further, considering the trends in declining precipitation and rising temperatures during the last 20 years in the study region, our results add to the accumulated knowledge of the possible impact of climate change on intraspecific genetic diversity [51, 52].

## Conclusion

The combined approach of quantitative trait differentiation (P_ST_) and F_ST_ estimates highlighted the adaptive role of flower phenology, growth and fitness-related traits in the adaptation of populations of *E. sativa* to local conditions in the eastern Mediterranean. Thus, the results support the hypothesis that phenotypic divergence evolved as a result of past diversifying selection, possibly imposed by an aridity gradient. However, to better infer on the impact of specific climatic variable(s) on phenotypic differentiation a more elaborated geographical sampling is needed, covering a higher number of populations. Further research involving reciprocal transplanting experiments at the different sites, as well as under controlled conditions, are also needed to confirm and evaluate the evolutionary resilience of populations from different environments [17, 53], which are especially important in the scope of global climate change [54].

## Methods

### The studied sites and plant material

Leaves and seeds of *Eruca sativa* were sampled from three different sites along the Jordan Valley: Ein Gev (EG), Bet Shean (BS) and Sartaba (SA), representing an aridity gradient of Mediterranean, semi-arid and desert habitats, respectively (Fig. 1 and Additional file 1: Table S1). Dr. Jotham Ziffer-Berger, the associate curator at the herbarium of the Steinhardt Museum of Natural History, Tel Aviv University (https://smnh.tau.ac.il/en/research-at-smnh-2/the-museum-collections/herbarium-collection/) verified the identification of *E. sativa*, where a voucher specimen was deposited (TELA 2918). The three populations represent the different genetic clusters of *E. sativa* along the investigated geographical range [25]. For the genetic analysis (below), we collected leaf samples in January 2016. Leaf samples were collected from 22–27 plants of each population (Table 1) and stored at − 80 °C before DNA extraction. At the end of the growing season, in May 2016, seeds were collected on the same day from 30 plants at each site. An equal number of seeds per plant were pooled together to create one seed lot per population. The seed lots were used in the common garden experiments, described below. The studied protocols complied with relevant institutional, national, and international guidelines and legislation. Plant material was collected under a permit granted by the Israel Nature and National Parks Protection Authority (permit #2016/41368).

Abiotic environmental parameters that distinguish the three natural habitats, i.e., annual rainfall, average temperature during the growing season and soil salinity [25] were gathered from the Geographic Information System Center Database, Hebrew University of Jerusalem, and from Westberg et al. [25]. In addition, information on yearly amount of rainfall and average daily temperatures at the peak of growing season (January) were gathered from meteorological stations located nearby the investigated natural sites, allowing inferences about climatic conditions over the last 20 years.

### Common garden experiments

Seeds of each of the three populations were placed in Petri dishes on water-saturated filter paper and germinated in a growth chamber at 25 °C with an 8/16 h day/night photoperiod. Four-day-old seedlings were transferred to germination trays, and after 2 weeks were transplanted to either the experimental field site at the Agricultural Research Organization (ARO) (Additional file 1: Table S1), or into 2 L pots containing a mixture of 50% peat, 30% tuff and 20% perlite (Shacham, Israel). The potted seedlings (one seedling per pot) were placed in a roof-covered net-house. Potted plants were irrigated daily (150 mL/day) with an automatic drip irrigation system, conditions previously found to be optimal for growth of *E. sativa* in this potting soil mixture [25]. Both the field and net-house experiments included 20 plants from each population, arranged in a randomized order. The two experiments took place between December 2017 and March 2018.

In the field experiment, plants were planted 1 m apart. The monthly distribution of rainfall between December 2017 to March 2018 was 57.6, 179.9, 85.0 and 5.5 mm per month, respectively. At this site, which is in a Mediterranean environment (Additional file 1: Table S1), plants were grown on well-drained sandy soil, different from the substrates and climatic conditions in their natural habitats [25]. Under such edaphic conditions, and as rain events were not distributed evenly, it was necessary to irrigate the plants once every three days in order to ensure plants survived during droughts longer than 1 week. The plants were monitored daily, and the onset and end of flowering were determined as days after germination. Duration of flowering was determined as the number of days elapsed between the onset and end of flowering (determined when the last flowers wilted).

In the net-house pots experiment, 10 plants were harvested on the day of flowering and roots and shoots were separated. Roots were washed in water and the dry weight (g) of the roots and shoots of each harvested plant was determined after drying for 48 h at 70 °C. The remaining 10 plants of each population were allowed to continue to grow and used for the assessment of plant fitness, by counting the total number of fruits and determining the total seed weight of each plant; seeds were cleaned from plant debris by passing them through sieves (up to a mesh-size of 1.0 mm).

The variation of the three phenological traits, below- and above-ground biomass, and fitness-related traits, were assessed by box-plot. The Kaplan Meier survival curve analysis was applied to assess differences in the three phenological traits, as these trait variables are time-to-event data. The Kaplan Meier analysis was implemented based on Cox proportion hazards regression models by treating population as a fixed factor. Further, a log-rank (Mantel–Cox) test was used to test for significance of the results. In addition, one way ANOVA was applied to test for significant difference in root and shoot weights and fitness-related traits (IBM SPSS Statistics Inc., ver. 23) by keeping population as a fixed factor.

### DNA extraction and AFLP analysis

DNA from the sampled leaves was extracted with the DNeasy Plant Mini Kit (QIAGEN, Hilden, Germany). AFLP analysis was carried out using the methodology described by Westberg et al. [25]. Restriction and ligation of DNA to adapters was carried out simultaneously (EcoRI, 5′-CTCGTAGACTGCGTACC-3′/5′-A ATTGGTACGCAGTC-3′; MseI, 5′-GACGATGAGTCCTGAG-3′/5′-TACTCAGG ACTCT-3′) at 23 °C for 14 h. The pre-selective amplification was performed with the primers E01 (5′-GACTGCGTACCAATTCA-3′) and M02 (5′-GATGAGTCCTGAGTAAC-3′), and the primers E37 (E01 + CG), E39 (E01 + GA), and E45 (E01 + TG) were used in the selective amplification in combination with M54 (M02 + CT) and M55 (M02 + GA), to provide six different combinations. The AFLP products were separated on an ABI 3100 automated sequencer (Applied Biosystems, ABI, Weiterstadt, Germany) as a multiplex of three primer combinations labeled with fluorescent dyes (6-FAM, NED, and HEX; Applied Biosystems, ABI), together with an internal size standard labeled with ROX (ROX 500; Applied Biosystems, ABI). Electropherograms were scored automatically with Genmarker 1.75 (SoftGenetics, State College, PA) and corrected manually.

The banding patterns obtained from the AFLP analysis were scored as present (1) or absent (0), each of which was treated with an independent character (Additional file 2). POPGENE version 1.32 [55] was used to calculate the different genetic diversity parameters: number of polymorphic loci (NPL), percentage polymorphic loci (PPL), Nei’s gene diversity (H), Shannon’s information index (I) and gene flow. The SPSS program was used to calculate confidence intervals (95%) for genetic diversity indices by 1000 bootstraps. The partitioning of genetic variability was calculated by analysis of molecular variance (AMOVA) using the GenAlEx program, version 6.3 [56].

The F_ST_ genetic differentiation coefficient proposed by Wright [57] was further used to test differentiation at the genetic level. F_ST_ was calculated using the AFLP-SURV program (https://www2.ulb.ac.be/sciences/lagev/aflp-surv.html) which implements the procedure proposed by Lynch and Milligan [58]. In this program, Bayesian statistics with non-uniform prior distribution were applied, considering a random mating population [fixed inbreeding coefficient (F_IS_) value as 0]; 500 permutations were applied with 95% confidence intervals.

### Estimation of quantitative trait differentiation (PST)

Following Morente-López et al. [16], P_ST_ was calculated using the equation: *P*_*ST*_ = *c/h*^*2*^ × *VC/(c/h*^*2*^ × *VC* + *2* × *VC*_*error*_*)*, in which *VC* is the phenotypic variance component of the population, *VC*_*error*_ is the residual variance, *h*^*2*^ is the proportion of phenotypic variance that is due to additive genetic effects within population, and *c* is the proportion of total variance that is presumed to be due to the additive genetic effect among populations. The *c/h*^*2*^ range of 0 to 2 was used for estimation of P_ST_, which is generally smaller than 1.0, since trait heritability is usually lower among populations than within populations [59]. Variance components (VC), used for estimation of P_ST_, were obtained from a random intercept of the generalized linear mixed model (GLMM). The GLMM model was implemented using the *lmer* function in the R-package *lme4* version 1.1-21 [60]. In GLMM, restricted maximum likelihood method (REML) was used for estimation of trait variance. GLMM was built using random intercept model, by considering the phenotypic parameter as a response variable and the population as predictor variable. The values of VC used for calculation of P_ST_ are given in Additional file 1: Table S4. P_ST_ confidence intervals (95%) were obtained by 1000 bootstrap iterations of the original data. Finally, for inferring past diversifying selection, the following criteria were used: (1) P_ST_ > F_ST_, for diversifying selection; (2) P_ST_ = F_ST_, for inferring that the trait differentiated neutrally; and (3) P_ST_ < F_ST_, for stabilizing selection [14].

## Supplementary Information

**Additional file 1: Table S1. **The investigated populations of *E. sativa*, their location, the annual precipitation, soil salinity and average temperature at the growing season. The conditions at the agricultural experimental field site (ARO) is also provided. **Table S2. **Analysis of molecular variance in populations of *E. sativa*. **Table S3.** Log-rank test applied on Kaplan–Meir survival curves for the measured phenological traits: the onset, duration and end of flowering (^*^P < 0.05). **Table S4.** Variance components of the tested phenological traits calculated using the generalized linear mixed model (GLMM). **Figure S1. **Annual rainfall (mm) and the average daily temperature in January at the natural sites of *E. sativa* in the 20 years that preceded the sampling. Data was obtained from meteorological stations located in vicinity to the studied sites.

**Additional file 2**. An Excel file with the list of the AFLP scored loci.

## Data Availability

All relevant data supporting our findings are presented in the article, additional information and additional Excel file. All laboratory protocols used in this study are available upon request.

## Abbreviations

AFLP: Amplified fragment length polymorphism
AMOVA: Analysis of molecular variance
BS: Bet Shean
CI: Confidence intervals
DAG: Days after germination
EG: Ein Gev
GLMM: Generalized linear mixed model
NPL: Number of polymorphic loci
PPL: Percentage polymorphic loci
SA: Sartaba
REML: Restricted maximum likelihood method
