# Supplementary material for: Phenotypic differentiation and diversifying selection in populations of Eruca sativa along an aridity gradient
Source: BMC Ecol Evol. 2022 Mar 30;22:40. doi: 10.1186/s12862-022-01996-w (PMC8966261; doi:10.1186/s12862-022-01996-w)
Supplement: Supplementary file 1 — Additional file 1: Table S1. The investigated populations of E. sativa, their location, the annual precipitation, soil salinity and average temperature at the growing season. The conditions at the agricultural experimental field site (ARO) is also provided. Table S2. Analysis of molecular variance in populations of E. sativa. Table S3. Log-rank test applied on Kaplan–Meir survival curves for the measured phenological traits: the onset, duration and end of flowering (*P < 0.05). Table S4. Variance components of the tested phenological traits calculated using the generalized linear mixed model (GLMM). Figure S1. Annual rainfall (mm) and the average daily temperature in January at the natural sites of E. sativa in the 20 years that preceded the sampling. Data was obtained from meteorological stations located in vicinity to the studied sites. [file 12862_2022_1996_MOESM1_ESM.docx]

**Phenotypic differentiation and diversifying selection in populations of *Eruca sativa* along an aridity gradient**

Prabodh Kumar Bajpai, Harel Weiss, Gony Dvir, Nir Hanin, Haggai Wasserstrom, Oz Barazani

**Additional Information**

**Table S1.** The investigated populations of *E. sativa*, their location, the annual precipitation, soil salinity and average temperature at the growing season. The conditions at the agricultural experimental field site (ARO) is also provided.

The average rainfall and temperatures were gathered from the Geographic Information System Center database (Hebrew University of Jerusalem) using coordinates of each population; values of electric conductivity (EC) from Westberg et al. [25].

|  | Coordinates (WGS84) | | Average annual rainfall (mm) | Soil salinity  (EC mS) | Temperature (°C)^*^ | Type of habitat^**^ |
| --- | --- | --- | --- | --- | --- | --- |
|  | Latitude (N) | Longitude (E) |  |  |  |  |
| Ein Gev (EG) | 32⁰ 46′ 44″ | 35⁰ 38′ 59″ | 356 | 0.16 | 12.3 | Mediterranean |
| Bet Shean (BS) | 32°30′ 04″ | 35° 30′ 38″ | 309 | 0.30 | 12.9 | Semi-arid |
| Sartaba (SA) | 32°04′ 49″ | 35° 29′ 46″ | 184 | 5.24 | 13.6 | Desert |
| ARO^***^ | 32° 46' 39'' | 35°39' 28'' | 401 | 0.07 | 13.8 | Mediterranean |

^*^Average daily temperature in January (winter).

^**^Based on Goldreich [20].

**^***^**Environmental conditions were determined in 2018, when the common garden experiment was conducted

**Table S2.** Analysis of molecular variance in populations of *E. sativa*

|  | DF | SSD | MSD | Estimated variance | Variance (%) |
| --- | --- | --- | --- | --- | --- |
| Within populations | 70 | 1051.557 | 15.022 | 15.022 | 93 |
| Among populations | 2 | 88.772 | 44.386 | 1.211 | 7 |

DF: degrees of freedom; SSD: sum of square deviation; MSD: mean square deviation

**Table S3.** Log-rank test applied on Kaplan-Meir survival curves for the measured phenological traits: the onset, duration and end of flowering (^*^*P*<0.05).

|  |  | Onset | Duration | End |  |
| --- | --- | --- | --- | --- | --- |
|  | df | χ^2^ | χ^2^ | χ^2^ |  |
| Population | 2 | 13.68^*^ | 10.89^*^ | 0.07 | |

**Table S4.** Variance components of the tested phenological traits calculated using the generalized linear mixed model (GLMM)

| Trait | Group | Variance | SD |
| --- | --- | --- | --- |
| Onset of flowering | Population(intercept) | 19.96 | 4.467 |
|  | Residual | 31.04 | 5.572 |
| Flowering duration | Population(intercept) | 12.11 | 3.479 |
|  | Residual | 47.93 | 6.923 |
| End of flowering | Population(intercept) | 0.1543 | 0.3928 |
|  | Residual | 10.7612 | 3.2804 |

**Figure S1.** Annual rainfall (mm) and the average daily temperature in January at the natural sites of *E. sativa* in the 20 years that preceded the sampling. Data was obtained from meteorological stations located in vicinity to the studied sites.
